# Good communication was valued as more important than accessibility according to 707 Nordic primary care patients: a report from the QUALICOPC study

**DOI:** 10.1080/02813432.2021.1928837

**Published:** 2021-05-27

**Authors:** Torunn Bjerve Eide, Jørund Straand, Anja Maria Braend

**Affiliations:** a Department. of general practice, Institute of Health and Society, University of Oslo, Oslo, Norway; b General Practice Research Unit (AFE), Department of general practice, Institute of Health and Society, University of Oslo, Norway

**Keywords:** General practice, QUALICOPC, delivery of health care, patient preference, Nordic countries

## Abstract

**Objective:**

To explore Nordic patients’ ranking of the importance of different aspects of general practice.

**Design:**

Patients ranked the importance of 47 statements reflecting five quality domains: communication, involvement, accessibility, continuity, and comprehensiveness.

**Setting:**

Nordic general practice.

**Subjects:**

Patients ≥18 years in general practitioners waiting rooms.

**Main outcome measures:**

Items rated as *important* or *very important* by ≥ 90% in all countries were identified. Associations with patient characteristics were analysed by logistic regression.

**Results:**

209 Danish, 175 Norwegian, 129 Finnish, 112 Swedish and 82 Icelandic patients responded. Ten statements were ranked as *important* or *very important* by ≥90% in each country. Six pertained to communication, three to patient involvement and one to the comprehensiveness of care. No items regarding accessibility or continuity exceeded the 90% limit. The item most frequently rated as *very important* was ‘I understand what the GP explains’’. Female patients were more likely to value personal treatment (OR = 2.9; 95%CI 1.5–5.5) and receiving instructions if things went wrong (1.7; 1.2–2.2). Older patients >65 years put less emphasis than those <35 on whether the GP takes them seriously (0.4; 0.3–0.5) and on the importance of instructions (0.5; 0.4–0.7). Patients with chronic diseases were less concerned (0.6; 0.4–0.8) with receiving instructions, but valued strongly that a GP knows when to refer (2.2; 1.5–3.3).

**Conclusion:**

Patients in all countries assigned high value to good communication. Availability was deemed important but came secondary to good communication.

**Implications:**

Organisational framework for general practice must allow for acceptable communication quality as well as availability.

**Key points**
- In order to identify relevant service areas for quality improvement in primary care, we aimed to increase knowledge of patient ranked importance of different dimensions of care.
- Nordic primary care patients valued good communication and involvement in decisions higher than accessibility to care.
- A singular focus on the access of care when developing services may not be in accordance with patient preferences.

## Introduction

In research on the quality of primary health care (PHC), the focus has over time shifted from *patient satisfaction* to *patient experiences* when using the services [1,2]. Patient satisfaction and patients’ experiences are commonly used as measures of quality in PHC research. This has given a better foundation for evaluating the quality of different aspects of the services. Research on PHC commonly reports descriptive data regarding patients’ experiences of e.g. ease of telephone access, waiting time for the appointment, consultation time, communication with the GP or continuity of care [3]. In the annual Commonwealth Funds evaluation of healthcare systems, Norwegian patients reported poorer experiences with their regular GPs than respondents from other countries in areas such as communication, user participation and consultation time, but they still reported higher general satisfaction with their GP than the European average [4]. This supports that a mere comparison of isolated quality measures for PHC across countries is not sufficient to evaluate whether change is warranted [5]. A recent systematic review points to several limitations of patient-related experience measures with regard to both validity and reliability, and questions whether such experience measures are applicable in international studies, as they are often specific to a setting, e.g. a health care system [6]. In order to ensure patient-centered care, we need to identify aspects of general practice that are most important for patients [7] thus ensuring that efforts to improve services are in line with patient priorities [8]. In studies listing different aspects of care that are all unequivocally positive, it is to be expected that patients will indicate all items as important.

In studies on patients’ experiences and satisfaction with PHC, most patients have generally positive views of the services [9,10]. In a European study involving 17,000 patients from ten countries [3], the patients were generally very positive towards the services, but a tendency towards less positive evaluation was found in countries where the GPs serve as gate-keepers for access to secondary care, as they do in the Scandinavian countries and the Netherlands. A review article from 2003 [11] found that patient characteristics (age, sex, economic status, family situation) were important determinants for how patients valued for instance accessibility, availability and organisation, with the most pronounced difference between younger and older patients. Younger patients placed greater importance on patient involvement and direct access to specialist healthcare, whereas older patients valued continuity of care higher [11]. A British study found that access in terms of opening hours and ease of making appointments were only modestly associated with the overall patient experience [12]. A Swedish study found that health care professionals believed patients to be less satisfied with primary care services than what the patients reported themselves [13].

To ensure expedient use of both human and financial resources we need a broad knowledge foundation that includes knowledge of what patients deem as the most important aspects of care. In a Norwegian qualitative study from 2000, patients indicated that communication with the GP was more important than easy access and short waiting time [14], but we have otherwise little knowledge from a Nordic setting regarding patient-ranked importance of different aspects of PHC quality. Even though there are some significant differences in the organisation of PHC between the Nordic countries, they all have tax-financed, equitable, high-quality healthcare services with general practice in a central role [15,16]. It is therefore likely that Nordic patients have somewhat similar expectations to their GPs.

In the current study, we aim to explore Nordic patients’ evaluation of the importance of different aspects of general practice, and to analyse possible associations between patients’ preferences and patients’ characteristics.

## Material and methods

### Questionnaires

Our data originate from the study Quality and Costs of Primary Care in Europe (QUALICOPC) which aimed to evaluate the performance of primary care systems in Europe in terms of quality, equity and costs [17]. The QUALICOPC Partner Consortium, led by the Netherlands Institute for Health Services Research (NIVEL), developed the questionnaires. One patient per participating GP answered a Patient Values Questionnaire (PVQ), designed to explore which aspects of general practice were rated as most important by patients, independently of their current consultation reason. The questions were derived from existing questionnaires, validated in three consensus rounds, followed by a pilot study, before the final revision. The QUALICOPC study and the development of questionnaires are described in further details elsewhere [18,19].

### Questionnaire items

The patients assessed 47 items reflecting various aspects of the contact with their GP in terms of importance (not important, somewhat important, important, very important) (Table 2). Fifteen of the items pertained to communication, sixteen patient involvement, seven accessibility, six continuity of care and three pertained to the comprehensiveness of services. The sorting of items into these five domains was done according to the validation procedure of the QUALICOPC questionnaires [18].

**Table 2.** Percentage of patients that answered *important* or *very important* to each questionnaire item (domains as defined by QUALICOPC consortium).

*Domain*	Total (*N* = 707)	Norway (*N* = 175)	Denmark (*N* = 209)	Sweden (*N* = 112)	Finland (*N* = 129)	Iceland (*N* = 82)
*Communication*						
Reception desk is polite and helpful	88.7	93.6	82.5	93.8	86.8	90.1
GP avoids disturbance by calls etc.	67.9	69.4	65.2	86.4	50.4	74.4
GP is polite	89.8	94.2	85.1	96.4	85.3	89.7
GP asks questions about my health problem	**95.1**	**98.8**	**92.3**	**98.2**	**94.5**	**91.3**
I understand what the GP explains	**97.3**	**98.8**	**95.2**	**99.1**	**98.4**	**95.0**
GP makes eye contact	84.5	88.4	88.9	88.3	82.7	62
GP listens attentively	**96.3**	**98.2**	**95.2**	**99.1**	**93.8**	**95.0**
GP is not prejudiced(age, gender, religion, culture)	85.1	88.9	81.2	89.1	77.5	93.8
GP treats me as a person, not just medical problem	**94.7**	**95.9**	**93.9**	**96.4**	**90.7**	**98.8**
GP is respectful	87.5	87.7	85.8	90.0	85.3	91.3
GP takes me seriously	**97**	**99.4**	**94.7**	**99.1**	**95.3**	**97.5**
GP understands me	**95.7**	**97.7**	**92.8**	**99.1**	**93.7**	**97.5**
GP asks if I have questions	84.1	88.7	75.5	92.7	82.4	87.5
GP asks if I have understood everything	83.9	85.9	79.9	91.6	86.3	86.3
I am honest and do not feel embarrassed	92.8	95.4	89.8	95.4	93.0	91.0
*Involvement*						
GP involves me in decision making	91	95.4	87.9	96.4	87.6	87.5
I feel better able to cope after GP visit	93.1	94.8	91.8	96.4	94.5	86.3
I have prepared by symptom diary or prepared questions	69.4	62.6	71.7	90.2	60.9	63
GP asks if I have questions	84.1	88.7	75.5	92.7	82.4	87.5
I can bring family/friend to the consultation	55.2	48.8	58.7	71.4	44.9	53.1
I keep to my appointment (with my doctor)	**96.4**	**98.8**	**96.6**	**99.1**	**93.0**	**90.9**
GP asks how I prefer to be treated	77.5	83.8	65.4	74.2	78.7	97.4
GP gives me additional info about health problem	58.3	62.6	54.4	67.0	55.8	51.2
GP informs me about reliable sources of info	46.4	47.1	42.2	56.5	33.9	62.0
I tell the GP what I want to discuss in consultation	79.5	77.3	73.2	87.2	78.1	92.5
I am prepared to ask questions and take notes	57.7	57.6	49.3	78.0	52.8	60.0
I am open about use of other treatments	78.6	83.7	78.5	74.1	78.1	74.0
GP gives me all test results	82.2	90.1	77.4	89.9	65.9	93.7
GP offers telephone or mail contact if further questions	78.0	91.2	65.2	86.2	70.3	83.5
Clear instructions what to do if things go wrong	**94.4**	**98.3**	**90.9**	**98.2**	**92.2**	**93.6**
I adhere to agreed treatment	**96.5**	**98.3**	**96.6**	**96.3**	**95.3**	**94.9**
I inform the GP how treatment works out	81.6	90.0	77.1	86.8	69.6	87.3
*Accessibility*						
GP does not give me feeling of time pressure	93.6	98.8	90.8	96.4	88.3	93.8
Practice has extensive opening hours	60.2	56.2	37.7	93.8	57.8	82.7
I can get appointment easily	90.1	93.1	79.1	98.2	94.6	93.7
I know how to get night/weekend services	80.7	66.7	80.2	89.1	87.6	89.9
Practice is close to where I live or work	69.4	69.6	57.8	81.8	75.2	72.2
I can see another doctor if I think it is necessary	68.8	71.8	62.1	86.5	50.0	85.3
Short waiting time on the phone	78.3	82.4	62.3	93.7	81.4	85
*Continuity*						
Medical records at hand	83.3	89.9	68.5	83.3	92.2	80.2
GP knows about my medical background	91.9	93.0	92.3	94.6	87.5	91.4
GP knows about my living condition	68.2	67.8	69.9	64.9	66.7	71.6
I don’t have to tell reception about my problems	62.0	74.6	56.3	61.6	51.6	67.1
I know which GP I will see	81.4	89.6	77.3	75.9	80.5	83.5
GP is aware of my personal background	57.1	60.7	58.5	59.5	37.5	73.8
*Comprehensiveness*						
GP knows when to refer	**96.7**	**97.1**	**95.6**	**97.2**	**96.1**	**98.7**
GP asks about possible other problems	69	73.4	54.1	79.5	73.4	75.9
Psychosocial problems can be discussed	83.1	88.9	85.8	90.6	64.8	83.3

**Bold**: ≥90% in all five countries answered *important* or *very important*.

### Sample

Patients ≥18 years who had booked appointments on a randomly selected day with a GP participating in the QUALICOPC study were approached in the GP’s waiting room by either medical students or nurse assistants who were engaged as field workers for the study and invited to answer an anonymous questionnaire. In Sweden and Denmark, random national samples of GPs were invited to participate in the QUALICOPC study. In Iceland, all GPs were invited. Finland employed a mixed procedure of random sampling and selected GPs. Norway used convenience sampling within formal and informal GP networks. Ten patients per GP were included in the QUALICOPC study, of which one was randomly assigned to answer the PVQ. All questionnaires were answered anonymously. Data collection took place from 2011 to 2013.

### Statistical analyses

Chi-squared test was used to check for differences in sociodemographic characteristics. In order to identify qualities that were universally considered of high importance, we identified the questionnaire items where 90% or more of patients in each country answered that they rated the indicated quality as *important* or *very important.* We used multiple logistic regression to identify associations with patients’ gender, age, health and level of education. Fisher’s exact test revealed that for six of the ten items there were significant inter-country differences in patients’ responses. We, therefore, progressed with a General Estimating Equations model, in order to correct for the clustered nature of the material. We did a Bonferroni correction to adjust for multiple analyses, defining significant p-values as *p* < 0.05/10 = 0.005. Since Bonferroni is a conservative correction, results with *p* < 0.05 are also highlighted. Results are given as percentages, or as odds ratios (OR) with 95% confidence intervals (CI). To visualize differences between patients’ ratings in different countries, we identified the top ten items per country that received the highest percentage of the answer *very important*. Analyses were done by SPSS Statistics 26 and Stata 16.1.

## Results

The material for the study comprised questionnaires from 707 patients; 209 Danish, 175 Norwegian, 129 Finnish, 112 Swedish, and 82 Icelandic. Table 1 shows demographic data of the study patients. Patients’ mean age varied from 49.0 (Norway) to 58.3 (Finland). There were fewer female participants from Iceland than from the other countries (52% versus 61-63%). Significantly more patients in Finland than in the other countries reported that they did not have very good health, a larger proportion had a chronic disease and their average level of education was also lower than reported from the other countries.

**Table 1.** Demographic data of patients participating in the study.

	Total *N* (%)	Norway	Denmark	Sweden	Finland	Iceland	*p*-value
Total *N* (%)	707 (100)	175 (24.8)	209 (29.6)	112 (15.8)	129 (18.2)	82 (11.6)	
Age							.001
Mean (range)^a^	53 (18–96)	49 (18–92)	52.8 (18–87)	55.6 (20–91)	57.3 (18–96)	52.2 (18–87)	
Female	430 (61.2)	110 (63.2)	129 (61.7)	70 (63.1)	79 (61.2)	42 (52.5)	.450
Own health^b^							
Very good	114 (16.4)	33 (19.1)	49 (23.8)	17 (15.5)	4 (3.2)	11 (13.6)	<.001
Good	333 (47.9)	84 (48.6)	91 (44.2)	54 (49.1)	56 (44.8)	48 (59.3)	.186
Fair	205 (29.5)	45 (26.0)	56 (27.2)	31 (28.2)	56 (44.8)	17 (21.0)	.002
Poor	43 (6.2)	11 (6.4)	10 (4.9)	8 (7.3)	9 (7.2)	5 (6.2)	.905
Chronic disease^c^	351 (49.6)	83 (48.3)	98 (46.9)	56 (50.5)	79 (63.2)	35 (43.8)	.049
Level of education^d^							
Primary education	192(27.2)	20 (11.4)	51 (24.4)	27 (24.1)	71 (55)	23 (28.0)	<.001
Secondary education	226 (32)	79 (45.1)	26 (12.4)	44 (39.3)	42 (32.6)	35 (42.7)	<.001
Higher education	270 (38.2)	73 (41.7)	124 (59.3)	39 (34.8)	16 (12.4)	18 (22.0)	<.001

Missing data: ^a^4; ^b^12; ^c^10; ^d^19.

Table 2 shows the percentages of patients from each country that rated each item as *important* or *very important*. For ten of the 47 questionnaire items, 90% or more of the patients in each country answered *important* or *very important* (bold print in Table 2). Six of these ten items pertained to the communication with the GP, three items pertained to patient involvement and one to comprehensiveness. No items regarding accessibility or continuity of care reached the 90% limit in all countries. However, for the item ‘‘I can get an appointment easily’’, 99% of Norwegian patients answered *important* or *very important*, and in Sweden, Iceland and Finland more than 90% answered similarly. However, only 79% of the Danish patients answered that this was important or very important, hence this item did not reach our predefined limit for further analyses.

Six items did not reach the 90% limit in each country, but the mean when all countries were analysed together was still above 90% (Table 2). Two of these items were found in the accessibility domain, whereas there was one each in the domains communication, involvement and continuity.

Table 3 shows the top ten items per country for the answer *very important.* The item most frequently rated as *very important* was ‘I understand what the GP explains’. This item came highest for three countries (Sweden 78%, Finland 68% and Iceland 75%) and second highest for Norway (71%) and Denmark (68%). In Norway, the item ‘The GP takes me seriously’ was most valued (73%), and in Denmark the item ‘I keep to my appointment’ (with my GP*)* received the highest percentage of *very important* (71%).

**Table 3.** Top ten questionnaire items that patients rated as *very important* per country.

	Norway (%)	Denmark (%)	Sweden (%)	Finland (%)	Iceland (%)
1	GP takes me seriously (73.1)	I keep to my appointment (71.1)	I understand what the GP explains (77.5)	I understand what the GP explains (67.7)	I understand what the GP explains (75.0)
2	I understand what the GP explains (70.9)	I understand what the GP explains (67.6)	GP knows when to refer (72.9)	GP knows when to refer (64.8)	GP takes me seriously (70.0)
3	GP knows when to refer (67.1)	GP knows when to refer (64.6)	GP takes me seriously (70.0)	Medical rec-ords at hand (55.0)	GP under-stands me (69.6)
4	GP involves me in decision making (64.2)	GP takes me seriously (63.3)	GP understands me (62.6)	GP takes me seriously (54.3)	GP knows when to refer (69.6)
5	Clear instructions if things go wrong (60.9)	GP treats me as a person, not just a medical problem (61.7)	GP listens attentively (62.2)	I feel better able to cope after a visit (52.3)	GP treats me as a person, not just a medical problem (67.5)
6	GP understands me (60.2)	I adhere to the agreed treatment (61.2)	GP does not give a feeling of time pressure (60.7)	GP understands me (52.0)	GP knows my medical background (63.0)
7	I keep to my appointment (59.9)	GP knows my medical background (60.8)	GP asks questions about health problem (60.4)	GP knows my medical background (51.6)	GP does not give a feeling of time pressure (62.5)
8	I adhere to the agreed treatment (58.1)	GP understands me (57.5)	I keep to my appointment (60.4)	I keep to my appointment (51.3)	GP is not prejudiced (62.5)
9	GP does not give a feeling of time pressure (57.6)	I feel better able to cope after a visit (57.0)	I adhere to the agreed treatment (58.7)	I adhere to the agreed treatment (50.0)	GP asks how I prefer to be treated (60.3)
10	GP treats me as a person, not just a medical problem (57.3)	GP listens attentively (56.3)	GP is polite (57.7)	Clear instructions if things go wrong (48.4)	I can get an appointment easily (59.5)

Percentages in brackets.

Items that were rated as *important* or *very important* by 90% or more in all five countries were further analysed with multiple regression analyses (Table 4). For five of these ten items, there were no significant associations with the patients’ gender, age, health status or level of education. Female patients were more likely than males to highly value that the GP treated them as a person and not only as a medical problem (OR 2.9; 95% CI 1.5–5.5), that they kept to their appointments with their doctor (OR 2.9; 1.8–4.7) and that they received instructions regarding what to do if things should go wrong (OR 1.7; 1.2–2.2). Older patients >65 years put less emphasis than the youngest patients <35 years on whether the GP took them seriously (OR 0.4; 0.3–0.5), and whether they received instructions on what to do if things went wrong (OR 0.5; 0.4–0.7). Patients who rated their own health as good put less value in keeping to their own appointment (OR 0.4; 0.2–0.7), and patients with a chronic disease were less concerned with receiving instructions (OR 0.6; 0.4–0.8) but valued highly that the GP knew when to refer them to secondary care (OR 2.2; 1.5–3.3).

**Table 4.** Associations between patient characteristics and quality of care related items rated as important/very important by ≥90% of patients in all Nordic countries.

	GP asks questions OR (95% CI)	Understand GP’s explanation OR (95% CI)	GP listens attentively OR (95% CI)	GP takes me seriously OR (95% CI)	GP understands me OR (95% CI)	GP treats me as a person OR (95% CI)	I keep to my appointment OR (95% CI)	Instructions what to do if things go wrong OR (95% CI)	I adhere to the treatment OR (95% CI)	GP knows when to refer OR (95% CI)
Gender (ref male)	1.6 (1.0–2.7)^a^	2.9 (1.1–7.6)^a^	2.4 (1.4–4.1)	2.3 (0.7–7.8)	1.3 (0.7–2.3)	**2.9 (1.5–5.5)^b^**	**2.9 (1.8–4.7)^b^**	**1.7 (1.2–2.2)^b^**	1.2 (0.7–1–9)	0.4 (0.5–4.9)
Age (ref <35)										
35–65	0.8 (0.2–3.1)	0.8 (0.1–4.4)	0.2 (0.0–0.9)^a^	0.5 (0.2–1.2)	2.0 (0.5–3.2)	**1.9 (1.2–3.0)^b^**	1.8 (0.6–5.3)	0.7 (0.4–1.2)	1.4 (0.3–6.7)	1.6 (0.6–3.9)
>65	0.8 (0.3–1.9)	0.7 (0.1–4.6)	0.2 (0.0–0.9)^a^	**0.4 (0.3–0.5)^b^**	1.2 (0.5–3.2)	1.8 (1.0–3.1)	2.2 (0.6–8.0)	**0.5 (0.4–0.7)^b^**	1.4 (0.3–6.5)	0.5 (0.2–1.7)
Own health (ref good)										
Poor	0.4 (0.2–0.8)	0.6 (0.3–1.1)	0.4 (0.1–0.9)^a^	0.6 (0.2–1.8)	1.2 (0.5–3.1)	1.1 (0.5–2.5)	**0.4 (0.2–0.7)^b^**	0.9 (0.4–2.1)	0.9 (0.3–3.0)	0.8 (0.5–1.4)
Chronic disease (ref no)	1.7 (0.8–3.5)	1.3 (0.5–1.3)	1.5 (0.6–3.7)	1.9 (0.7–4.9)	0.6 (0.2–1.7)	0.8 (0.3–2.0)	1.6 (1.1–2.6)^a^	**0.6 (0.4–0.8)^b^**	1.4 (0.9–2.1)	**2.2 (1.5–3.3)^b^**
Level of education (ref primary school)										
High-school/college	1.4 (0.6–3.3)	0.5 (0.1–1.7)	0.7 (0.2–1.9)	1.2 (0.6–2.7)	1.5 (0.9–2.3)	1.0 (0.4–2.6)	1.1 (0.8–1.5)	1.6 (0.9–2.9)	0.6 (0.2–1.7)	1.0 (0.7–1.5)
Higher education	1.4 (0.9–2.2)	**0.4 (0.3–0.4)^b^**	0.6 (0.4–0.9)^a^	1.1 ( 0.8–1.6)	0.9 (0.4–1.8)	1.0 (0.6–1.5)	1.2 (0.8–2.0)	1.2 (0.8–1.8)	0.8 (0.6–1.2)	1.3 (0.6–2.6)

^a^*p* < .05; ^b^*p* < .005 (Bonferroni correction).

General estimating equations logistic regression analyses. Associations showed as odds ratios (ORs) with 95% confidence intervals (CI). Statistically significant findings (*p* < .005) are indicated in **bold print**.

## Discussion

To provide patient-centered care, we need to know what patients prefer, not make assumptions about their preferences. With the intention to determine aspects of care that should have priority for further PHC quality improvement, we have identified preferences universal to patients in all Nordic countries. Features related to communication with the GP and patient involvement were ranked as most important by the study patients. For all the countries, ‘I understand what the GP explains’ was among the top three answers in the *very important* category. No items from the accessibility domain reached this top three list.

In our study, the clear majority of items reaching the 90% limit in all countries were in the communication domain (six of 15 items in this domain reached the limit). The item ‘The GP takes me seriously’ was among the top four in all the countries. This corresponds with a study of Swiss QUALICOPC data, which concluded that items related to communication/patient-centeredness and coordination/continuity of care were rated as more important than items related to access [20]. In the Europep study from 1999, performed in eight countries including Norway, Sweden and Denmark, the number one priority were that the GP should have enough time to listen, talk and explain, which is in line with our findings that patients place a very high value on the quality of communication with their GP [5]. Similarly, the factor most strongly associated with a positive patient experience in a recent British study was the GP’s interpersonal quality of care [12]. Patients who see a GP with strong empathic abilities have better clinical outcomes in fields as diverse as anxiety, diabetes and the common cold [21]. Patients of empathic doctors experience better enablement, and also report higher satisfaction with the services [21]. With increasing focus on the productivity of care, this is important to bear in mind. It corresponds with our finding that items relating to doctor-patient communication are highly valued by patients.

People with a chronic disease or poor health are expected to be frequent users of health services, and it is thus reasonable to pay attention to which aspects of the services they deem as important. Somewhat surprisingly, people with self-evaluated poor health put less value into their own involvement in terms of keeping to their GP appointment. People with chronic diseases were less concerned with receiving instructions on what to do if things went wrong – maybe because they already know how to handle their chronic disease? They did, however, value higher than others that the GP knows when to refer – maybe they have experiences to the contrary.

In our study, none of the items regarding accessibility were deemed as important/very important by more than 90% in each country. Looking at the top ten answers for each country in the *very important* category, the only items from the accessibility domain was ‘The GP does not give me a feeling of time pressure’ (ranked No. 5 in Sweden, No. 7 in Iceland and No. 9 in Norway), and ‘I can get an appointment easily’ (No. 10 in Iceland). This corresponds with a British study from 2018, where the overall patient satisfaction with PHC contacts was only moderately associated with accessibility [12]. Only 88% of the Finnish patients found it important not to be given a feeling of time pressure. This may be related to the fact that Finnish GPs estimate an average of 24 min per patient consultation [16], hence the patients may be used to sufficient duration of consultations. On the other hand, Swedish GPs estimate the same mean duration of consultations as the Finnish, whereas 96% of Swedish patients rate the lack of time pressure as very important. A more plausible interpretation may therefore be cultural differences in expectations when contacting the health services. In a recent study on patient enablement, cultural factors that pertained to the national culture, rather than inter-country differences in health care systems, were found to be associated with patient enablement after a GP consultation [22]. It is possible that such national cultural differences also affect other aspects of healthcare.

No items in the continuity domain reached the 90% limit for all countries in our study, in spite of evidence that continuity of care is associated with better patient satisfaction, better adherence to medical advice and also lower mortality [23,24]. Although continuity is increasingly recognised by GPs as an important feature of PHC, its’ importance may be less obvious for patients in a time with electronic patient records and easily accessible medical information online. In the UK, there has been a strong focus on shortening waiting time for consultations, and this has come at the expense of continuity of care with less possibility to see the same GP every time [25]. Patients give high priority to access in situations they perceive as urgent [5], but in other situations they are willing to wait longer in order to see a doctor they know [25]. Patients’ preferences vary according to the reason for seeking healthcare, and it seems like patients’ main preference is to receive *appropriate* services [25]. In our study, over 90% of the patients in all countries except Denmark, gave high importance to the item ‘I can get an appointment easily’. It seems clear that patients do want easy access to appointments, but it is equally important that the doctor they meet has time and focus to listen and explain properly, and show empathy for and acceptance of their patients. A cross-sectional British study identified areas for improvement in general practice [26]. The authors commented that while better access to out-of-hours care would likely improve patient satisfaction, it was not likely that a shorter waiting time in regular general practice would improve patient satisfaction. They also pointed to patient empowerment as an area where improvement will most likely enhance patient satisfaction.

Quality improvement measures for the organization of primary care should take general patient preferences into account. Patient-centered care may be defined as compassionate and empathetic care that is responsive to the needs, values, and preferences of each individual patient [27]. This increases patients’ satisfaction and is associated with better adherence to medication and better self-management of chronic diseases [27,28]. A focus limited for instance to accessibility may, according to findings both in our and in previous studies, not be in accordance with patient preferences if it comes at the expense of good communication with the GP. Accessibility of primary care is easier to measure than the quality of communication, patient-centeredness or continuity of care, and may therefore receive more attention than what is warranted. The framework of general practice must allow for all aspects of care, and it is important that efforts to improve one aspect do not cause repression of the others.

### Strength and weaknesses

The QUALICOPC study was based on validated questionnaires, providing a good opportunity to obtain comparable data from different countries [18]. In all the Nordic countries except Sweden, the GPs and their patients were recruited from the whole country and both urban and rural areas. The recruitment procedure was not quite identical in all countries, which somewhat reduces the comparability of the data. In Finland, a majority of GPs were recruited from public health centers as opposed to occupational health centers [29], the latter being an alternative access point to primary care for many Finnish people [30]. There may be demographical differences between the users of public healthcare centers and the users of other primary care services, possibly mirrored in the fact that Finnish participants had poorer health and a lower level of education than the patients from the other countries.

We consider study participants to be representative of people who are users of general practice, but since we recruited the patients in the GPs’ waiting rooms, we cannot draw conclusions about preferences among persons who do not visit their GP. However, a large majority (in Norway 70%) of the population visit their GP each year, hence a GP waiting room population may be seen as representative of the general population. A qualitative methodology could give more in-depth information on how patients value the different dimensions of their contact with primary care.

## Conclusions/implications

Nordic patients highly valued good communication with their GPs and also involvement in decision making. The framework of general practice should support and endorse the qualities of care that are valued by patients, in line with the idea of patient-centered care. When developing primary care services, it should be kept in mind that a singular focus on one aspect of services may result in a poorer quality of other, equally important, service dimensions.

### Ethics approval and consent to participate

The QUALICOPC study was presented to the relevant ethic committees in the Nordic countries. The study was approved by the Danish Data Agency, the Ethical Committee of the Pirkanmaa Hospital District in Finland, the Regional Ethical Review Board of Linköping in Sweden (Dnr 2011/481-31; Dnr 2013/120-32) and the Icelandic National Bioethics Committee. The Regional Committee for Medical and Health Research Ethics in South-Eastern Norway concluded that their approval was not required for this study.

## Data Availability

The raw data used in this study is the property of the international QUALICOPC consortium, and is not available for publication by the authors. The data is available upon reasonable request.
